# In-hospital outcomes of catheter ablation in atrial arrhythmias: a nationwide analysis of 2,901 patients with adult congenital heart disease compared to 787,995 without

**DOI:** 10.1007/s00392-025-02614-7

**Published:** 2025-02-24

**Authors:** Sebastian Grundmann, Klaus Kaier, Alexander Maier, Jonathan Rilinger, Johannes Steinfurt, Brigitte Stiller, Dirk Westermann, Constantin von zur Mühlen, Markus Jäckel

**Affiliations:** 1 Department of Cardiology and Angiology, Faculty of Medicine, University Heart Center Freiburg - Bad Krozingen, University of Freiburg, Hugstetter Strasse 55, 79106 Freiburg, Germany; 2 Center for Big Data Analysis in Cardiology (CeBAC), Department of Cardiology and Angiology, Faculty of Medicine, University Heart Center Freiburg - Bad Krozingen, University of Freiburg, Freiburg, Germany; 3 Institute of Medical Biometry and Statistics, Faculty of Medicine and Medical Center, University of Freiburg, Freiburg, Germany; 4 Department of Congenital Heart Defects and Pediatric Cardiology, Medical Center, University Heart Center Freiburg - Bad Krozingen, University of Freiburg, Freiburg, Germany

**Keywords:** Congenital heart disease, Atrial arrhythmia, Ablation, Safety, Cardiac tamponade

## Abstract

**Background:**

Advances in pediatric cardiology and congenital heart surgery have increased the adult population with congenital heart disease (CHD), now facing long-term complications like atrial arrhythmias. Given the limited data and safety concerns in this unique and vulnerable patient group, this study analyzes in-hospital outcomes of atrial catheter ablation in CHD patients versus non-CHD patients from a German nationwide real-world registry.

**Methods:**

Using health records, all atrial catheter ablation procedures in Germany from 2008 to 2021 were analyzed. After adjustment for confounders, safety performance endpoints were compared between patients with and without CHD.

**Results:**

From 2008 to 2021, 790,896 patients underwent right or left atrial catheter ablation in Germany. Of these, 1004 patients were classified as simple CHD, 1,054 patients as moderate CHD and 843 patients as complex CHD. Age at time of procedure was lower with increasing complexity of the CHD. Atypical atrial flutter (5.5% vs. 21.8%; *p* < 0.001) and other atrial tachycardias (21.2% vs. 42.2%; *p* < 0.001) occurred more often in patients with complex CHD compared to patients without. Combined ablation in both atria was more often performed in complex CHD. Despite higher complexity, in-hospital mortality (< 0.2%) and other investigated complications were rare. After adjustment for baseline characteristics, type of arrhythmia and ablation location, the relative risk for serious adverse events (combination of mortality, stroke, intracerebral bleeding or pericardiocentesis) did not show a significant difference for patients with CHD.

**Conclusion:**

Even in patients with CHD, complications are rare and after adjustment, no differences were identified concerning serious adverse events. Therefore, an ablation should not be generally avoided in patients with CHD due to concerns about complications although an individualized evaluation of the anatomy must be taken into account.

**Graphical Abstract:**

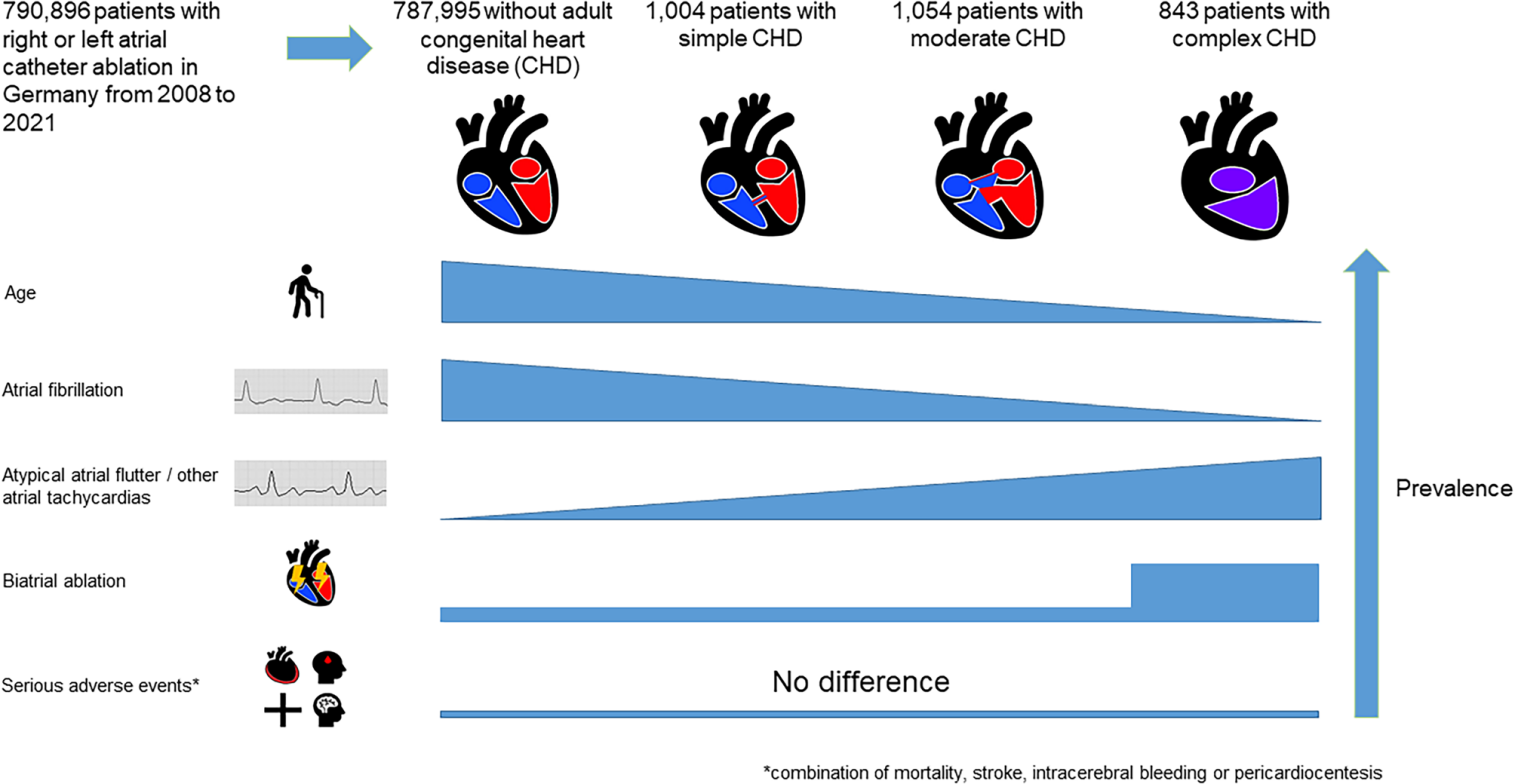

**Supplementary Information:**

The online version contains supplementary material available at 10.1007/s00392-025-02614-7.

## Background

Due to significant advancements in pediatric cardiology and surgical techniques for correcting congenital heart defects over the past decades, the majority of patients with congenital heart defects (CHDs) now survive into adulthood. As a result, there is a growing population of survivors and aging patients with repaired or palliated CHD [1]. Consequently, long-term complications, such as heart rhythm disturbances, are now occurring within this patient population and have become a significant source of morbidity. Projections suggest that 50% of 20 year-olds with CHD will experience atrial tachyarrhythmia at some point during their lifetime [2]. Additionally, the incidence increases steadily with age and is associated with a doubling of the risk of adverse events [2]. Abnormal anatomy, postoperative scarring, and systemic factors create a unique substrate for arrhythmia generation [3]. Many CHD patients do not respond satisfactory to antiarrhythmic pharmacologic therapy for CHD, which has generally been discouraging, leading most centers and recent guidelines to favor interventional approaches instead [4].

The number of catheter ablation procedures increased steadily in the last two decades and advances in ablation technologies improved outcome in these patients [5]. The most common types of atrial arrhythmias are intra-atrial reentry tachycardias with an acute success rate of more than 80% [6]. Atrial fibrillation is also increasing exponentially in this aging population [7]. However, due to the heterogeneity of the individuals with CHD, data on ablation strategy and outcomes in CHD are very rare and safety concerns of catheter ablation because of the structural abnormalities in these patients frequently preclude interventional approval.

To examine the safety of catheter ablation in this special patient collective, we analyzed in-hospital outcomes and complications in patients who underwent an atrial ablation procedure and had CHD coded as a comorbidity in their electronic health record, compared to patients without such an additional documentation of CHD, in Germany from 2008 to 2021.

## Methods

### Data acquisition

Since 2005, comprehensive data on all hospitalizations in Germany have been accessible for research purposes through the diagnosis-related group (DRG) statistics maintained by the Research Data Center of the Federal Bureau of Statistics (DESTATIS). The database encompasses a complete collection of hospitalization records in German hospitals reimbursed under the DRG system. Diagnoses and procedures in the database are encoded according to the ICD-10-GM (German Modification) and the Operation and Procedure Classification (OPS) systems. The Research Data Center is structured to provide aggregated data outcomes rather than individual patient records, eliminating the need for ethics committee approval and informed consent. DESTATIS ensures that all provided data summaries are anonymized, safeguarding patient confidentiality.

### Diagnoses and outcomes definitions

From this database, we extracted data of all adult patients aged 18 and older, hospitalized between 2008 and 2021 with right (OPS 883520; 883,530; 883,540; 883,580; 8835a0; 8835b0; 8835c0; 8835d0; 8835g0 and left (OPS 883523; 883,533; 883,543; 883,583; 88,359; 8835a3; 8835b3; 8835c3; 8835d3; 8835g3;883,525; 883,535; 883,545; 8835a5; 8835b5; 8835c5; 8835g5) atrial catheter ablation.

Patients with CHD were identified and classified as simple, moderate, and complex CHD according to Bethesda disease complexity classification as described before [8, 9]. Specifically, simple CHD included ventricular septal defect, patent ductus arteriosus, and congenital valvular lesions. Moderate CHD included tetralogy of Fallot including pulmonary atresia with ventricular septal defect, Ebstein anomaly, aortic coarctation / interrupted aortic arch, atrioventricular septal defect, and partial anomalous pulmonary venous drainage. Complex CHD included univentricular heart, Eisenmenger syndrome, transposition of the great arteries, and other complex CHD lesions (truncus arteriosus communis and total anomalous pulmonary venous connection).

The primary outcomes were serious adverse events (combination of mortality, stroke, intracerebral bleeding, or pericardiocentesis). Secondary outcomes were in-hospital mortality, stroke, intracerebral hemorrhage, pericardiocentesis, mechanical ventilation > 48 h, major bleeding (defined as the need for transfusion of > 5 units of red blood cells), acute kidney injury, length of hospital stay, and reimbursement.

ICD and OPS codes for analysis of comorbidities and complications during in-hospital stay are shown on Supplemental Table 1. In-hospital mortality and length of hospital stay were part of DESTATIS’ main set of variables.

**Table 1:** Baseline characteristics of all patients in Germany 2008–2021 with catheter ablation of atrial arrhythmias and no / simple / moderate / complex congenital heart disease

	No CHD (N = 787,995)	Simple CHD (N = 1,004)	Moderate CHD (N = 1,054)	Complex CHD (N = 843)	p value simple vs no CHD	p value moderate vs no CHD	p value complex vs no CHD
Female	316,452 (40.2%)	330 (32.9%)	520 (49.3%)	299 (35.5%)	< 0.001	< 0.001	0.005
Age	63.39 (13.20)	51.64 (15.65)	47.66 (15.48)	37.38 (11.95)	< 0.001	< 0.001	< 0.001
CCI	0.81 (1.25)	1.03 (1.24)	0.93 (1.09)	0.99 (0.96)	< 0.001	0.002	< 0.001
CHA2DS2VASC	2.35 (1.55)	1.61 (1.33)	1.48 (1.22)	0.99 (0.93)	< 0.001	< 0.001	< 0.001
Congestive heart failure	172,183 (21.9%)	328 (32.7%)	353 (33.5%)	291 (34.5%)	< 0.001	< 0.001	< 0.001
NYHA III/IV	69,605 (8.8%)	131 (13.0%)	110 (10.4%)	82 (9.7%)	< 0.001	0.067	0.361
Coronary artery disease	181,867 (23.1%)	152 (15.1%)	88 (8.3%)	33 (3.9%)	< 0.001	< 0.001	< 0.001
Arterial Hypertension	480,556 (61.0%)	420 (41.8%)	323 (30.6%)	122 (14.5%)	< 0.001	< 0.001	< 0.001
Previous myocardial infarction	24,242 (3.1%)	21 (2.1%)	9 (0.9%)	5 (0.6%)	0.071	< 0.001	< 0.001
Previous ACVB or valve surgery	42,026 (5.3%)	89 (8.9%)	194 (18.4%)	75 (8.9%)	< 0.001	< 0.001	< 0.001
Peripheral artery disease	13,254 (1.7%)	9 (0.9%)	6 (0.6%)	1–2 (≤ 0.2%)	0.053	0.005	< 0.001
Carotid Disease	3,550 (0.5%)	1–2 (≤ 0.2%)	1–2 (≤ 0.2%)	1–2 (≤ 0.2%)	0.235	0.206	0.354
COPD	32,705 (4.2%)	26 (2.6%)	28 (2.7%)	4 (0.5%)	0.013	0.015	< 0.001
Pulmonary Hypertension	19,891 (2.5%)	72 (7.2%)	74 (7.0%)	51 (6.0%)	< 0.001	< 0.001	< 0.001
Chronic kidney disease	84,727 (10.8%)	81 (8.1%)	80 (7.6%)	36 (4.3%)	0.006	0.002	< 0.001
Diabetes	106,482 (13.5%)	79 (7.9%)	59 (5.6%)	35 (4.2%)	< 0.001	< 0.001	< 0.001
Previous stroke	2,992 (0.4%)	4 (0.4%)	4 (0.4%)	8 (0.9%)	0.005	0.999	0.007
Hemiplegia or paraplegia	527 (0.1%)	1–2 (≤ 0.2%)	1–2 (≤ 0.2%)	1–2 (≤ 0.2%)	0.106	0.124	0.057
Dementia	1,588 (0.2%)	1–2 (≤ 0.2%)	1–2 (≤ 0.2%)	0 (0%)	1.000	0.933	0.812
Connective tissue disease	6,987 (0.9%)	1–2 (≤ 0.2%)	3 (0.3%)	3 (0.4%)	0.020	0.037	0.100
Peptic ulcer disease	1,176 (0.1%)	0 (0%)	1–2 (≤ 0.2%)	1–2 (≤ 0.2%)	0.221	0.733	0.509
Mild liver disease	5,303 (0.7%)	19 (1.9%)	31 (2.9%)	17 (2.0%)	< 0.001	< 0.001	< 0.001
Moderate/severe liver disease	629 (0.1%)	1–2 (≤ 0.2%)	1–2 (≤ 0.2%)	3 (0.4%)	0.181	0.207	0.110
Cancer	5,186 (0.7%)	0 (0%)	5 (0.5%)	1–2 (≤ 0.2%)	0.010	0.461	0.018
Metastatic solid tumor	780 (0.1%)	1–2 (≤ 0.2%)	0 (0%)	0 (0%)	0.313	0.307	0.361

Chi^2^ (for categorical variables) or t tests (for continuous variables). P value is reported in bold if difference is significant (p < 0.05). Data are given as mean and ± standard deviation or number of patients (percent of all patients per group). For case numbers of 1 and 2, the numbers are not explicitly reported by DESTATIS due to data protection regulations. P values are given for N = 2

*CCI* Charlson comorbidity index, *COPD* Chronic obstructive pulmonary disease, *CHD* Congenital heart disease. Data are given as mean and ± standard deviation or percent of all patients per group

### Statistical analysis

Data are given as n (%), mean ± standard deviation if not stated otherwise. Complication rates were compared using either chi^2^ (for categorical variables) or t tests (for continuous variables). Since the patients were not randomly assigned to the two energy sources analyzed (RF vs CB), confounder adjusted logistic regression models were carried out. As the number of potential confounders was high, the double-selection lasso logit regression model was applied as described by Belloni et al., a lasso logistic regression model is fitted and odds ratios are reported [10]. As double-selection algorithm, we applied the adaptive lasso (least absolute shrinkage and selection operator) for variable selection. A major advantage of the adaptive lasso is its oracle property, which improves the selection of relevant variables [11]. Cluster robust standard errors at the hospital level were specified to accommodate the correlation of error terms among patients treated at the same hospital. All baseline characteristics, as well as the type of arrhythmia, the ablation location, and the energy source were included as potential confounders to control for varying patient profiles.

Two-sided p values are given, and statistical significance was considered as p value < 0.05. No adjustments for multiple testing were done.

All analyses were carried out using Stata 18 (StataCorp, College Station, Texas, USA) and Prism (version 8, GraphPad Software, San Diego, USA). Figures were created using PowerPoint (version 2019, Microsoft, Redmond, USA).

## Results

### Number of procedures

**Fig. 1:**
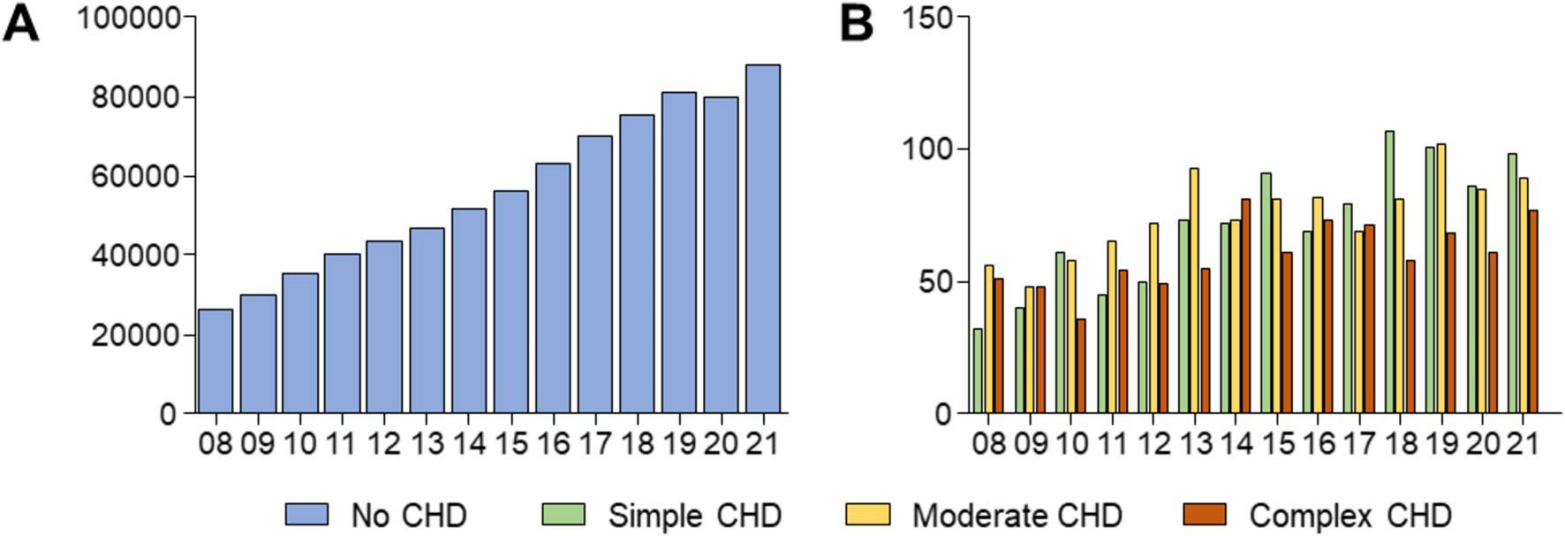
Procedure numbers of atrial ablations in patients without **A** simple, moderate, and complex **B** congenital heart disease (CHD) in Germany from 2008 to 2021

From 2008 to 2021, 790,896 patients underwent right or left atrial catheter ablation in Germany. Of these, 2901 patients had an additional DRG-code of CHD in their electronic health record submitted by the health care provider for reimbursement purposes, with 1004 patients having simple CHD, 1,054 patients had moderate CHD and 843 patients complex CHD. Numbers of procedures increased steadily from 2008 to 2021 in all patients (Fig. 1).

### Baseline characteristics

Of all patients undergoing catheter ablation, mean age was 63 years and 40.2% were female. Age was lower with increasing complexity of the CHD and we found higher rates of congestive heart failure and pulmonary hypertension in patients with CHD. Common age-related diseases as coronary artery disease, arterial hypertension, COPD, chronic kidney disease, and diabetes were more frequent in patients without CHD (Table 1). For the classification of the specific congenital heart diseases, see Table 2.

**Table 2:** Classification of the specific congenital heart diseases of all patients in Germany 2008–2021 with catheter ablation of atrial arrhythmias and simple / moderate / complex congenital heart disease

**Simple CHD**	
Ventricular septal defect	290 (28.9%)
Patent ductus arteriosus	37 (3.7%)
Congenital valvular lesions	716 (71.3%)
**Moderate CHD**	
Tetralogy of Fallot (including pulmonary atresia with ventricular septal defect)	377 (35.8%)
Ebstein anomaly	275 (26.1%)
Aortic coarctation / interrupted aortic arch	101 (9.6%)
Atrioventricular septal defect	177 (16.8%)
Partial anomalous pulmonary venous drainage	134 (12.7%)
**Complex CHD**	
Univentricular heart	231 (27.4%)
Eisenmenger syndrome	0 (0.0%)
Transposition of the great arteries	568 (67.4%)
Other complex CHD lesions (truncus arteriosus communis, total anomalous pulmonary venous connection)	36 (4.3%)

CHD Congenital heart disease

### Type of arrhythmia and ablation location

**Fig. 2:**
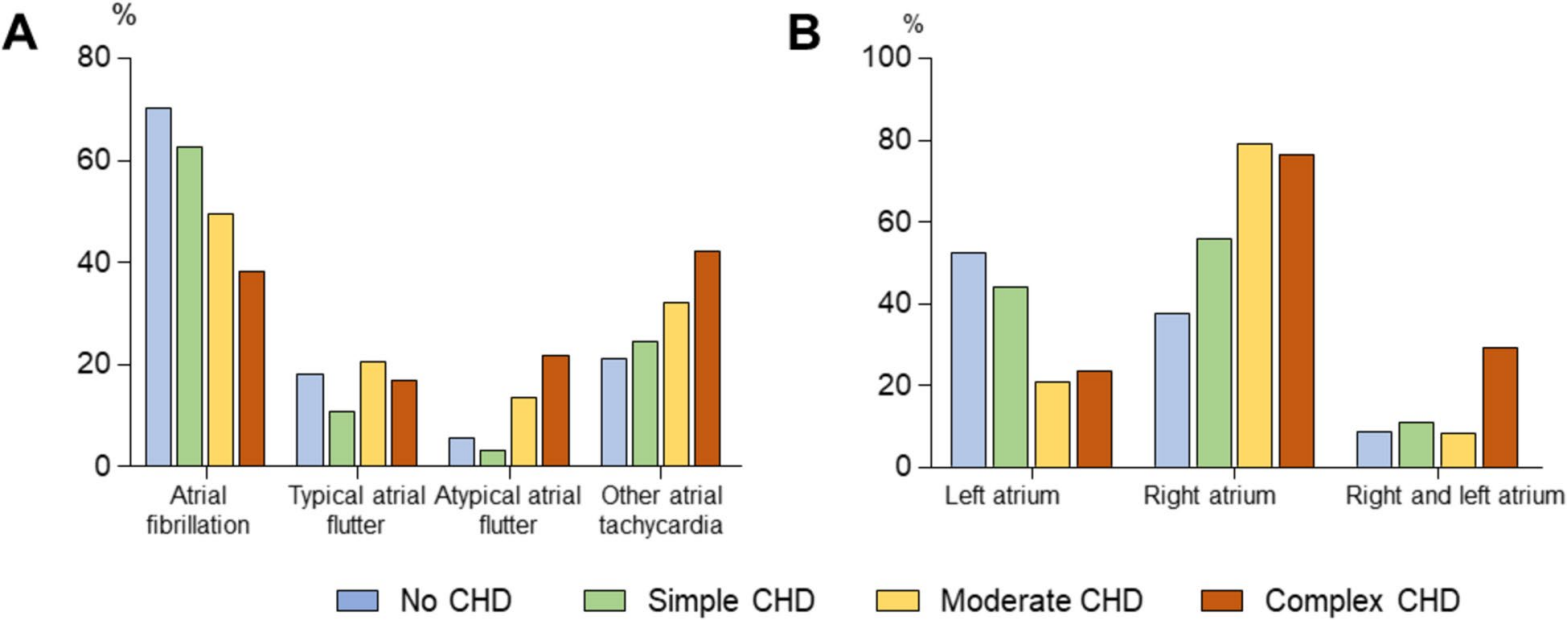
Type of arrhythmia **A** and ablation location **B** in patients without, simple, moderate, and complex congenital heart disease (CHD) in Germany from 2008 to 2021

Patients without CHD more often suffered from atrial fibrillation (70.3%), compared to patients with simple (62.7%), moderate (49.6%), and complex (38.2%) CHD (*p* < 0.001). Typical atrial flutter was diagnosed in 18.3% of all patients without CHD and in 10.7% / 20.7% / 17.1% of all patients with simple / moderate / complex CHD. Atypical atrial flutter occurred more often in patients with complex CHD (21.8%) compared to the patients with no / simple / moderate CHD (5.5% / 3.3% / 13.5%; *p* < 0.001). Other atrial tachycardias increased with increasing complexity of the CHD (no / simple / moderate / complex: 21.2% / 24.5% / 32.0% / 42.2%) (Fig. 2a, Supplemental Table 2).

Left atrial catheter ablation was more often performed in patients without CHD (no / simple / moderate / complex: 52.3% / 44.1% / 20.7% / 23.4%). Right atrial catheter ablation was performed in 47.7% of all patients without CHD and in 55.9% / 79.3% / 76.6% of all patients with simple / moderate / complex CHD (*p* < 0.001). Combined ablation in both atria was more often performed in complex CHD (29.1%) compared to no / simple / moderate CHD (8.6% / 11.1% / 8.1%; *p* < 0.001) (Fig. 2b, Supplemental Table 2).

### Safety outcome

Complication rates were low in all groups. Compared to patients without congenital heart disease, no differences were identified concerning serious adverse events (combination of mortality, stroke, intracerebral bleeding, or pericardiocentesis) in patients with simple / moderate or complex CHD. However, resuscitation was more often performed in patients with moderate and complex CHD compared to patients without CHD (0.7% / 0.9% vs 0.3%; *p* < 0.05). In-hospital mortality did not differ (no / simple / moderate / complex CHD: 0.1% / 0.0% / 0.3% / ≤ 0.2%; all p values > 0.2). Ventilation for longer than 48 h was more often necessary in patients with simple / moderate / complex CHD compared to patients without (1.0% / 0.9% / 0.7% vs. 0.3%, *p* < 0.001) (Table 3). Pericardiocentesis was more often necessary in patients with simple CHD compared to patients without CHD (1.3% vs 0.7%; *p* = 0.013), while it was rarely documented in patients with complex CHD (no CHD: 0.7% versus complex CHD: ≤ 0.2%; *p* = 0.131).

**Table 3:** Outcome characteristics of all patients in Germany 2008–2021 with catheter ablation of atrial arrhythmias and no / simple / moderate / complex congenital heart disease. Serious adverse events = in-hospital mortality, stroke, Intracerebral bleeding, or pericardiocentesis

	no CHD (N = 787,995)	Simple CHD (N = 1,004)	Moderate CHD (N = 1,054)	Complex CHD (N = 843)	p value simple vs no	p value moderate vs no	p value complex vs no
Serious adverse events	8,221 (1.0%)	14 (1.4%)	10 (0.9%)	5 (0.6%)	0.274	0.763	0.198
In-hospital mortality	1,115 (0.1%)	0 (0%)	3 (0.3%)	1–2 (≤ 0.2%)	0.233	0.217	0.460
Pericardiocentesis	5,182 (0.7%)	13 (1.3%)	3 (0.3%)	1–2 (≤ 0.2%)	0.013	0.134	0.131
Stroke	2,122 (0.3%)	1–2 (≤ 0.2%)	4 (0.4%)	1–2 (≤ 0.2%)	0.669	0.476	0.858
Intracerebral bleeding	123 (0.0%)	0 (0%)	0 (0%)	0 (0%)	0.692	0.689	0.717
Ventilation > 48 h	2,251 (0.3%)	10 (1.0%)	9 (0.9%)	6 (0.7%)	< 0.001	0.001	0.021
Serious bleeding	1,469 (0.2%)	12 (1.2%)	4 (0.4%)	4 (0.5%)	< 0.001	0.147	0.053
Resuscitation	2,141 (0.3%)	3 (0.3%)	7 (0.7%)	8 (0.9%)	0.870	0.015	< 0.001
Acute kidney injury	8,179 (1.0%)	15 (1.5%)	8 (0.8%)	10 (1.2%)	0.154	0.372	0.6714
Length of Stay (days)	4.04 (4.75)	5.92 (7.64)	5.10 (5.64)	5.06 (5.08)	< 0.001	< 0.001	< 0.001
Reimbursement (€)	7391.67 (3921.37)	9108.67 (8050.67)	7556.41 (5385.10)	8005.26 (3450.79)	< 0.001	0.173	< 0.001

Chi^2^ (for categorical variables) or t tests (for continuous variables). P value is reported in bold if difference is significant (p < 0.05). Data are given as mean and ± standard deviation or number of patients (percent of all patients per group). For case numbers of 1 and 2, the numbers are not explicitly reported by DESTATIS due to data protection regulations. P values are given for N = 2

**Fig. 3:**
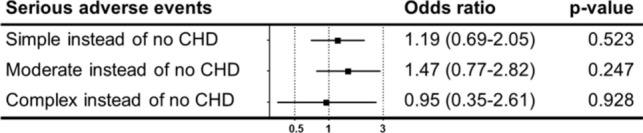
Analyses adjusted for baseline characteristics (see Table 1). Serious adverse events = in-hospital mortality, stroke, intracerebral bleeding, or pericardiocentesis

After adjustment for baseline characteristics, type of arrhythmia and ablation location (see Tables 1 and S2), the relative risk for serious adverse events did not show a significant difference for patients with CHD (Fig. 3).

## Discussion

We here report comprehensive and contemporary nationwide data sets on in-hospital safety outcomes of ablation procedures in patients with and without congenital heart disease performed in Germany between 2008 and 2021. Our findings suggest that catheter ablation is a safe treatment option for atrial arrhythmias in patients with CHD, even in patients with complex structural scenarios.

Arrhythmias are an important reason for hospitalization in ACHD patients and the main cause of emergency department or ICU admission [12]. With the increasing number of patients with congenital heart disease reaching adulthood and advanced age, they became a frequent cause of morbidity and mortality. Due to the specific anatomic and hemodynamic pre-conditions, the clinical consequences of arrhythmias are often amplified in ACHD patients and even relatively harmless atrial arrhythmias, which are usually well controllable in patients without CHD, can result in significant hemodynamic compromise and severe symptoms. Among the different types of arrhythmias, postoperative atrial arrhythmias are the most common in this patient population and about 15% of ACHD patients will experience a supraventricular tachycardia (SVT) during the course of disease, with a much higher rate in patients with complex lesions [2]. At the same time, atrial arrhythmias are associated with a severe increase in cardiac death especially early after arrhythmia onset, and show a strong correlation with stroke and heart failure [2, 13]. While current guidelines mostly recommend a trial of antiarrhythmic medication as the primary approach for most atrial arrhythmias, the long-term use of such drugs is associated with severe adverse effects and many CHD patients present contraindications to this approach due to pre-existing conduction abnormalities or an already impaired systemic ventricular function [14]. Therefore, catheter ablation is recommended as an alternative to long-term medical therapy for symptomatic sustained recurrent SVT or atrial macro-reentry tachycardia, especially in patients with simple CHD lesions [14]. However, increased procedural complexity, reduced efficacy, and especially safety concerns currently limit the implementation of catheter ablation in more patients with moderate and complex CHD scenarios.

While the specific anatomic substrate naturally differs between patients depending on the specific CHD and the mode of repair or palliation, causes of atrial arrhythmias can be summarized under increased volume and pressure load of the atria as well as postsurgical anatomic substrates due to scars, patches, and sutures. This results in a significant difference in patients with atrial arrhythmias unrelated to congenital heart disease also in our data set. While atrial fibrillation was more present in patients without CHD (70.3%) but also in patients with simple CHD lesions (62.7%), this percentage decreased with increasing complexity of CHD, when atypical atrial macro-reentry tachycardias and other atrial tachycardias became more frequent. In good correspondence with this finding, our results document an increasingly extensive interventional approach in complex CHD scenarios, with 29.1% of patients requiring combined left and right atrial procedures.

Somewhat surprisingly, and despite these more extensive and complex procedures, our data do not show a significant difference in short-term in-hospital adverse events in patients with congenital heart disease of all levels of complexity in comparison to patients without CHD after adjustment for baseline characteristics, type of arrhythmic arrhythmia and ablation location. Although we detected a higher rate of cardiopulmonary resuscitation during hospital admission in patients with moderate and complex CHD scenarios, this did not result in a higher rate of in-hospital mortality. In patients with simple CHD, serious bleeding events and pericardiocentesis were more frequent than in patients without CHD, while we did not detect a statistically significant difference in the other CHD subgroups. While this could be a chance finding related to an overall low event rate, complex CHD patients are generally treated in more experienced specialized arrhythmia centers, potentially resulting in a lower operator-related complication rate. The higher rate of prolonged ventilation in CHD patients is most likely related to the high percentage of procedures done under general anesthesia compared to the treated population without CHD.

Our findings are a good correspondence with the results of a recently published international multicenter registry study on catheter ablation for atrial fibrillation in 240 patients across the spectrum of ACHD-severity, with 25 patients having severely complex structural disease [15]. While the authors found a statistically higher number of complications in the severe complexity group, this was primarily driven by a single death in this subgroup, while no mortality occurred in the simple and moderate complexity subgroups.

## Limitations

Compared to this study and other previous studies in this field, which mostly focused on small registries on the efficacy of catheter ablation in specific CHD lesions, our type of analysis has specific limitations but also advantages [16–18]. It is the largest published cohort of ACHD patients treated by catheter ablation of atrial arrhythmias, comprising all patients treated in Germany between 2008 and 2021, the last year for which data were available for analysis. As diagnosis, comorbidities and procedures are primarily coded for reimbursement purposes, and complexity of the underlying pathology as well as a larger resource utilization due to adverse events generally results in a higher reimbursement, there is a significant incentive to code procedural complications compared to traditional registries. However, this analysis is based on administrative data, which is known to be frequently incomplete, especially for complex conditions such as CHD, and coding errors are possible [19]. This is potentially reflected by the lower than expected number of procedures per year, suggesting underreporting of CHD cases and a possible dilution of the “no CHD group” especially by patients with simple CHD lesions. However, this is less likely for patients with complex CHD scenarios, which frequently trigger higher reimbursement. In addition, this does not preclude our conclusions of an overall low in-hospital adverse event rate following ablation of atrial arrhythmias across the whole spectrum of ACHD. As the available data only comprise information collected during the initial hospital admission and aggregation with follow-up visits and hospital admissions is not possible due to data safety concerns in Germany, our data are limited to the reported short-term adverse events rates and do not allow any conclusions about long-term complications or procedural efficacy. Additionally, whether a surgical correction of congenital heart disease was performed and how this correction was carried out cannot be determined or queried from the available data, which may influence the risk of complications.

## Conclusion

In summary, our analysis shows a significant change in treatment practice of atrial arrhythmia in patients with adult congenital heart disease over time, mirroring the increased use of catheter ablation in patients without congenital heart disease in the recent years. Although the primary treatment strategy for atrial arrhythmias has to be chosen in an individualized manner in this heterogeneous patient population, our data demonstrate an overall low risk of serious procedural complications also in complex ACHD patients and show that catheter ablation does not have to be avoided in this patient population due to safety concerns. Our data further support the initiation of clinical registries that allow more detailed analysis of efficacy and long-term outcome of ablation procedures in ACHD patients.

## Supplementary Information

Below is the link to the electronic supplementary material.

Supplementary file1 (DOCX 32 KB)

## Data Availability

The datasets analyzed during the current study are not publicly available due to German data protection regulations but are available from the corresponding author on reasonable request.
