# Supplementary material for: In-hospital outcomes of catheter ablation in atrial arrhythmias: a nationwide analysis of 2,901 patients with adult congenital heart disease compared to 787,995 without
Source: Clin Res Cardiol. 2025 Feb 24;114(4):507–15. doi: 10.1007/s00392-025-02614-7 (PMC11946971; doi:10.1007/s00392-025-02614-7)
Supplement: Supplementary file 1 — Supplementary file1 (DOCX 32 KB) [file 392_2025_2614_MOESM1_ESM.docx]

| **Ablation location and type of arrhythmia** |  | |
| --- | --- | --- |
| Right atrial ablation  Left atrial ablation  Left atrial cryoballoon ablation | 883520; 883530; 883540; 883580; 8835a0; 8835b0; 8835c0; 8835d0; 8835g0  883523; 883533; 883543; 883583; 88359; 8835a3; 8835b3; 8835c3; 8835d3; 8835g3;883525; 883535; 883545; 8835a5; 8835b5; 8835c5; 8835g5  8835a3; 8835a5 | |
| Paroxysmal atrial fibrillation | I480 | |
| Persistent atrial fibrillation | I481 | |
| Atrial flutter, typical | I483 | |
| Atrial flutter, atypical  Other atrial tachycardia | I484  I471 | |
| **Classification of congenital heart disease (CHD)**  **Complex CHD**   \| Univentricular heart \| Q20.1, Q20.2, Q20.4, Q22.6, Q23.4, (Q22.0 and NOT Q21.0) \| \| --- \| --- \| \| Eisenmenger syndrome \| I27.8 and additional Q code, Q21.88 \| \| Transposition of the great arteries \| Q20.3, Q20.5, Q25.8, Q25.9 \| \| Other complex CHD lesions \| Q20.0, Q26.2 \|   **Moderate CHD** | | |
| Tetralogy of Fallot including pulmonary atresia with ventricular septal defect | | Q21.3, Q21.80, (Q22.0 and Q21.0) |
| Ebstein anomaly | | Q22.5 |
| Aortic coarctation/interrupted aortic arch | | Q25.1, Q25.2 |
| Atrioventricular septal defect | | Q21.2 |
| Partial anomalous pulmonary venous drainage  **Simple CHD** | | Q26.3, Q26.4 |
| Ventricular septal defect | | Q21.0 |
| Patent ductus arteriosus | | Q25.0 |
| Valvular lesions | | Q22.1, Q22.2, Q22.3, Q22.4, Q22.8, Q22.9, Q23.0, Q23.1, Q23.2, Q23.3 |
| **Baseline characteristics** |  | |
| Age |  | |
| Female |  | |
| Charlson Comorbidity Index (CCI) | see reference for complete ICD-10 list (1) | |
| Congestive heart failure | from CCI | |
| NYHA III / IV | I5013; I5014 | |
| coronary artery disease | I25 | |
| Hypertension | I10 | |
| Diabetes mellitus | E10; E11; E 12; E 13; E14 | |
| Previous stroke / TIA | I69 | |
| Previous Myocardial infarction | I252 | |
| Carotid disease | I652 | |
| COPD | J44 | |
| Pulmonary Hypertension | I27 | |
| Renal Disease | N18 | |
| Previous Stroke | I69 | |
| Hemiplegia or paraplegia | from CCI | |
| Dementia | from CCI | |
| Connective tissue disease | from CCI | |
| Peptic ulcer disease | from CCI | |
| Mild liver disease | from CCI | |
| Moderate / severe liver disease | from CCI | |
| Cancer | from CCI | |
| Metastatic solid tumor | from CCI | |
|  |  | |
| **Outcome** |  | |
| Pericardiocentesis | 1842; 53700; 81520; 53740; 53741; 53742; 53743; 53744; 53745 | |
| Stroke | I63 | |
| Intracerebral bleeding | I61 | |
| Ventilation >48h | 87181; 87182; 87183; 87184; 87185; 87186 | |
| Serious bleeding (>5 units RBC) | 88001; 88002; 88003; | |
| AKIN | I17 | |
| Length of Stay (days), mean SD | days | |
| Reimbursement, mean SD | SD | |

Table S1: Diagnosis and procedure codes used for this analysis. Diagnosis codes were coded according to the German Modification of the International Statistical Classification of Diseases and Related Health Problems 10th Revision (ICD-­‐10-­‐GM). Procedures were coded according to the German Procedure Classification (OPS)

|  | **noCKD (N=787,995)** | **Simple CHD (N=1,004)** | **Moderate CHD (N=1,054)** | **Complex CHD (N=843)** | **p-value simple vs no** | **p-value moderate vs no** | **p-value complex vs no** |
| --- | --- | --- | --- | --- | --- | --- | --- |
| Atrial fibrillation | 553,600 (70.3%) | 630 (62.7%) | 523 (49.6%) | 322 (38.2%) | <0.001 | <0.001 | <0.001 |
| Typical atrial flutter | 144,534 (18.3%) | 184 (18.3%) | 218 (20.7%) | 144 (17.1%) | 1.000 | **0.050** | 0.345 |
| Atypical atrial flutter | 43,553 (5.5%) | 107 (10.7%) | 142 (13.5%) | 184 (21.8%) | <0.001 | <0.001 | <0.001 |
| Other atrial tachycardia* | 167,220 (21.2%) | 246 (24.5%) | 337 (32.0%) | 356 (42.2%) | 0.011 | <0.001 | <0.001 |
| Cryoballoon ablation | 142,718 (18.1%) | 108 (10.8%) | 60 (5.7%) | 11 (1.3%) | <0.001 | <0.001 | <0.001 |
| Left atrial ablation | 412,327 (52.3%) | 443 (44.1%) | 218 (20.7%) | 197 (23.4%) | <0.001 | <0.001 | <0.001 |
| Right atrial ablation | 375,668 (47.7%) | 561 (55.9%) | 836 (79.3%) | 646 (76.6%) | <0.001 | <0.001 | <0.001 |
| Left and right atrial ablation | 68,129 (8.6%) | 111 (11.1%) | 85 (8.1%) | 245 (29.1%) | 0.001 | 0.502 | <0.001 |

Table S2: Catheter ablation characteristics of all patients in Germany 2008 - 2021 with catheter ablation of atrial arrhythmias and no / simple / moderate / complex congenital heart disease.

Chi2 (for categorical variables) or t-tests (for continuous variables). P value is reported in bold if difference is significant (p < 0.05). Data are given as mean and ± standard deviation or number of patients (percent of all patients per group)

*include: atrioventricular nodal reentrant tachycardia, atrioventricular reentrant tachycardia, focal atrial tachycardia

Literature Cited

1. Glasheen WP, Cordier T, Gumpina R, Haugh G, Davis J, Renda A. Charlson Comorbidity Index: ICD-9 Update and ICD-10 Translation. Am Health Drug Benefits 2019; 12(4):188–97.
